# Quercetin Loaded Cationic Solid Lipid Nanoparticles in a Mucoadhesive In Situ Gel—A Novel Intravesical Therapy Tackling Bladder Cancer

**DOI:** 10.3390/pharmaceutics14112527

**Published:** 2022-11-20

**Authors:** Sylvia Shawky, Shaimaa Makled, Ashraf Awaad, Nabila Boraie

**Affiliations:** 1 Department of pharmaceutics, Faculty of Pharmacy, Alexandria University, Alexandria 21521, Egypt; 2 Center of Excellence for Research in Regenerative Medicine Applications, Faculty of Medicine, Alexandria University, Alexandria 21500, Egypt

**Keywords:** bladder cancer, quercetin, coated SLNs, intravesical drug delivery, T-24 cell lines, mucoadhesive in situ gel

## Abstract

The study aim was to develop an intravesical delivery system of quercetin for bladder cancer management in order to improve drug efficacy, attain a controlled release profile and extend the residence time inside the bladder. Either uncoated or chitosan coated quercetin-loaded solid lipid nanoparticles (SLNs) were prepared and evaluated in terms of colloidal, morphological and thermal characteristics. Drug encapsulation efficiency and its release behaviour were assessed. Furthermore, cytotoxicity of SLNs on T-24 cells was evaluated. Ex vivo studies were carried out using bovine bladder mucosa. Spherical SLNs (≈250 nm) ensured good entrapment efficiencies (EE > 97%) and sustained drug release up to 142 h. Cytotoxicity profile revealed concentration-dependent toxicity recording an IC_50_ in the range of 1.6–8.9 μg/mL quercetin. SLNs were further dispersed in in situ hydrogels comprising poloxamer 407 (20%) with mucoadhesive polymers. In situ gels exhibited acceptable gelation temperatures (around 25 °C) and long erosion time (24–27 h). SLNs loaded gels displayed remarkably enhanced retention on bladder tissues relative to SLNs dispersions. Coated SLNs exhibited better penetration abilities compared to uncoated ones, while coated SLNs dispersed in gel (G_10_C-St-QCT-SLNs-2) showed the highest penetration up to 350 μm. Hence, G_10_C-St-QCT-SLNs-2 could be considered as a platform for intravesical quercetin delivery.

## 1. Introduction

Bladder cancer (BC) is among the top ten most frequent cancer types worldwide, where about 550,000 new cases are diagnosed with bladder cancer each year. It is considered as one of the most lethal cancer types, particularly in older males. BC is defined as abnormal cells proliferating in the lining of the bladder. It is classified into 5 stages (from 0 to IV) indicating the progression of the disease. In general, BC is divided into two types: non-muscle invasive bladder cancer (NMIBC) and muscle invasive bladder cancer (MIBC). At diagnosis, 70% of the cases would present with NMIBC, while in the other 30% the disease would have progressed to MIBC [1,2]. The standard therapy for NMIBC is the transurethral resection of bladder tumour (TURBT) in addition to intravesical chemotherapy and immunotherapy such as bacillus Calmette–Guérin (BCG), as well as systemic chemotherapy such as Mitomycin C (MMC) to have better control on tumour recurrence and progression [3].

Unfortunately, 70% of patients will experience tumour recurrence, with 25% advancing to MIBC within five years following TURBT [2]. The oral bioavailability of BC therapeutic agents is lowered by the gastric acid and the degradative hepatic enzymes, whereas the poorly vascularized urothelial cells would render systemic therapy less effective [4,5]. To enhance the therapeutic efficacy and achieve high drug levels within the bladder, higher doses of the systemically administered agents are required, which results in increased systemic adverse effects and high toxicity to healthy cells [3].

On the other hand, local administration ensures high concentrations of the therapeutic agents inside the target organs, while minimizing systemic side effects [4]. Because of the position and anatomy of the bladder, it is regarded as a perfect organ for intravesical drug delivery (IDD), which is defined as direct administration of the therapeutic agents into the bladder through a urethral catheter. Also, the presence of the permeability barrier of the bladder, with tightly packed umbrella cells and uroplakin plaques, hinders the systemic absorption of the therapeutic agents [5]. Therefore, maximum exposure of the bladder cancer cells to the chemotherapeutic agents with minimum systemic exposure could be achieved through IDD.

However, IDD has its own limitations as well. The presence of bladder permeability barrier (BPB), which hinders the penetration of the chemotherapeutic agents into the bladder tissues, in addition to frequent drug dilution and wash-out by urine diminish the efficacy of the intravesical therapy and may lead to therapeutic failure [6,7]. Thus, developing an efficient delivery system, that is able to overcome the limitations of IDD and achieve better control over bladder cancer, remains a challenge.

Quercetin (QCT) is a natural polyphenolic flavonoid found in variety of vegetables and fruits such as onions, apples and berries. It is chemically known as 3,3′,4′,5,7-pentahydroxyflavone (C_15_H_10_O_7_) [8]. Thanks to its good anticancer activity, low toxicity and wide accessibility, QCT was chosen as a model drug for intravesical therapy of bladder cancer. QCT has a wide array of pharmacological applications, including antioxidant [9], anti-inflammatory [10], antidiabetic [11] and anti-cancer properties [12]. Most importantly, QCT hinders the progression of many types of cancers, including cervical, colon, breast, liver, prostate and lung cancer [13]. Various mechanisms are thought to be responsible for the anticancer properties of QCT such as its ability to suppress enzymes involved in carcinogens activation and cellular signalling. Based on its ability to bind to cellular receptors and proteins, QCT exhibits a wide range of anticancer effects [14,15]. Furthermore, the combination of QCT with conventional chemotherapeutic agents displayed synergistic effects, which may further enhance the efficiency of the conventional chemotherapy [16]. Research carried out by Oršolić and colleagues showed that QCT has genotoxic and cytotoxic activity against T-24 cells (human bladder cancer cells). The chemotherapeutic effects of QCT were explained by its ability to expand the DNA damage of human bladder cancer cells, preventing cell propagation and colony formation [17]. However, clinical use of QCT is restricted by its poor water solubility and consequently poor bioavailability [18].

Smart drug carriers for IDD offer several advantages over conventional formulations, such as improved drug solubility and prolonged adhesion of the drug carriers to the urothelial surfaces, in addition to high penetration into malignant tissues. For instance, liposomes and polymeric nanoparticles have been widely used for IDD [19,20,21,22,23,24].

Solid lipid nanoparticles (SLNs) are considered as one of the most appropriate carriers for the delivery of lipophilic drugs as they ensure high drug encapsulation and controlled release properties, together with low toxicity. They have been widely employed for the delivery of several chemotherapeutic agents, such as docetaxel [25], etoposide [26] and doxorubicin [27], due to the high solubility of such lipophilic agents in the solid lipids [28].

Compared to liposomes, SLNs display improved entrapment efficiency for various lipophilic drugs and ensure higher stability of the loaded drugs due to the rigid core lipid matrix [3]. Furthermore, SLNs are considered safer drug carriers relative to polymeric nanoparticles as they can be synthesized using various techniques not involving the use of organic solvents [29]. Despite the abovementioned advantages of SLNs, they have scarcely been used for intravesical applications.

Improved response to intravesical chemotherapy was attained in patients who received positively charged ions with MMC compared to patients treated with MMC alone, which may be explained by the enhanced penetration of MMC into the bladder tissues in the presence of positively charged ions [30]. In spite of the promising outcomes, the implementation of electromotive drug administration into general practice is difficult and nearly restricted to the European academic centres [23]. Meanwhile, positively charged nanocarriers can improve drug delivery into urothelial layers without the need for such complex administration devices [23,24,31]. Being mucoadhesive, these cationic nanocarriers are able to efficiently attach to the mucosal surfaces, allowing prolonged contact of the formulations with the diseased cells and, hence, improved cellular uptake [5]. Furthermore, the use of cationic mucoadhesive polymers is believed to encourage paracellular transport through rearrangement of the tight junctions between cells and, hence, improves therapeutic agent penetration into the bladder wall [32].

Usually drug solutions are washed out by urination within 2 h following intravesical administration [33]. Administration of frequent doses of the drugs requires repeated catheterization which causes patient inconvenience and puts them at increased risk of infection. Therefore, development of an IDD system that can withstand wash-out by urine and remain inside the bladder for a prolonged period of time, allowing for continuous drug release, is essential. Various approaches have been developed to achieve this goal, among them the use of mucoadhesive thermosensitive in situ gels for IDD [34].

Thermosensitive polymers, including poloxamers, exist as liquid formulations during storage and administration and transform into gels inside the body. Previous literature reported the use of poloxamers for preparation of intravesical in situ gels with extended drug release properties [35]. Men K et al. introduced a composite system, wherein cationic nanocarriers were incorporated into an in situ poloxamer 407 (P407) based gel. The system offered extended retention of the formulations inside the bladder cavity as well as higher permeation into the bladder wall [31]. In another study, it was found that the use of P407 hydrogel together with mucoadhesive polymers led to remarkable effect in sustaining release of drug. However, the formulation exhibited very low gelation temperature which may cause a problem during handling and administration [36]. By optimizing gel composition, an easily administered in situ gel with rapid and efficacious adhesion to bladder tissues could be obtained.

Our aim was to develop a novel intravesical system for QCT delivery to enhance QCT performance in bladder cancer management and address the drawbacks of systemic drug delivery by direct local delivery into the bladder. Cationic QCT-loaded SLNs were synthesized and characterized in terms of their colloidal and thermal properties, encapsulation efficiency, and in vitro release behaviour. In addition, their in vitro cytotoxicity against the bladder cancer cell line was studied. Cationic SLNs were further incorporated into mucoadhesive in situ gels, as appropriate IDD systems, to extend the residence time and enhance treatment efficiency. The composite system of cationic nanoparticle and thermo-sensitive hydrogels was evaluated ex vivo in terms of its retention on bladder mucosa, penetration into the bladder wall, and safety on bladder tissues.

## 2. Materials and Methods

### 2.1. Materials

Quercetin was obtained from M/s Sisco Research Laboratories (Maharashtra, India). Precirol was a sample gift from Gattefossé (Saint Priest, France). Stearic acid was kindly provided by Pharco Pharmaceuticals Company (Alexandria, Egypt). Poloxamer188 (P188, Pluronic-F68TM) was obtained from BASF (Ludwigshafen, Germany). Tween 80 was purchased from Chemtech (Alexandria, Egypt). Chitosan (Cs) for coating (Mw 60–120 kDa, Degree of deacetylation 85%, viscosity 27 CPS) was purchased from Sigma-Aldrich (Steinheim, Germany). Poloxamer 407 (Pluronic F127) was a sample gift from Borg Pharmaceutical Companies (Alexandria, Egypt). Carbopol 974P (Cb) was a sample gift from Lubrizol (Oevel, Belgium). Chitosan for gel preparation (Mw 161.116, degree of deacetylation 93%) was obtained from Oxford Lab Chem (Mumbai, India). Hydroxy propyl methyl cellulose (HPMC) was purchased from Cairo Pharmaceuticals Co., (Cairo, Egypt). T-24: Urinary Bladder cancer cell line (transitional cell carcinoma) was obtained from Nawah Scientific Inc., (Cairo, Egypt). Dulbecco’s Modified Eagle Medium (DMEM) high glucose, Roswell Park Memorial Institute (RPMI) 1640 high glucose, Penicillin/streptomycin, Trypsin and EDTA were purchased from Lonza GmbH (Köln, Germany). Fetal bovine serum (FBS) was purchased from Gibco (Grand Island, NY, USA). Coumarin-6 dye (cou) and Sulforhodamine B (SRB) were purchased from Sigma-Aldrich (Steinheim, Germany). Trichloroacetic acid (TCA) was purchased from Merck (Kenilworth, NJ, USA). Tris (hydroxymethyl) aminomethane (TRIS) was obtained from Chem-Lab (Zedelgem, Belgium). All other used chemicals were of analytical grade.

### 2.2. Methods

#### 2.2.1. Preparation and In Vitro Characterization of SLNs

##### Solid Lipids Screening

The ability of different lipids to solubilize QCT was examined. For this purpose, solid lipids (50 mg) were heated 5–10 °C above their melting points. Then, QCT (5 mg) was added to the molten lipids and the mixture was kept under magnetic stirring until clear and homogeneous dispersion of QCT was obtained [37]. The lipids giving a clear homogeneous mixture of molten phase were selected for further studies.

##### Preparation of Plain, QCT-Loaded and Fluorescently Labelled SLNs

SLNs were prepared using probe ultrasonication [38], by melting different amounts of the solid lipids (precirol and stearic acid) with or without the drug (QCT) at 80 °C using a water bath. Three millilitres stabilizer’s solution, which was previously heated to 80 °C, was added dropwise to the molten lipids. The mixture was then ultra-sonicated at a power output of 60% amplitude for 10 min at 80 °C (Sonoplus HD 3100; BANDELIN, Berlin, Germany). At the end of the sonication step, a nanoemulsion was obtained. The obtained nanoemulsion was then added to 7 mL of cold water kept in an ice bath. To develop SLNs, the final mixture was ultra-sonicated at the same power output for 5 min immersed in the ice bath. As described in the results section, the impact of several formulation variables, such as stabilizer type, concentration and lipid to drug ratio was examined. For preparation of fluorescently labelled SLNs, used in the ex vivo studies, coumarin-6 (cou) was used instead of QCT and the aforementioned technique was again followed.

##### Preparation of Chitosan-Coated Nanoparticles

The prementioned nano-emulsion was added to a mixture of cold chitosan solution (0.025 or 0.1%) adjusted to a certain pH (5, 3.8 respectively) and cold water (1:1) kept in an ice bath. Then, the final mixture was ultrasonicated for 5 min.

##### Colloidal Characteristics and Morphology of Solid Lipid Nanoparticles

SLNs were analysed in terms of particle size (PS) and polydispersity index (PDI) by dynamic light scattering (DLS) using a Malvern Zetasizer^®^ (Zetasizer^®^ Nano ZS series DTS 1060, Malvern Instruments S.A, Worcestershire, UK) using a 4 mW He-Ne laser at 633 nm at a fixed angle (173°) at 25 °C. Zeta potential (ZP) was analysed at 25 °C using a cell current of 5 mA and voltage of 150 V. SLNs were properly diluted with deionized water (RI = 1.33) prior to analysis. Measurements were done in triplicates. In addition, after negative staining with uranyl acetate, the morphology of SLNs was studied using transmission electron microscopy (TEM), model JEM-100CX (JEOL, Tokyo, Japan).

##### Entrapment Efficiency and Drug Loading

Entrapment efficiency (EE) of QCT inside SLNs was indirectly assessed according to a modified centrifugal ultrafiltration method using Centrisart-I tube (MWCO 300 kDa, Sartorius AG, Goettingen, Germany). In brief, 2 mL of the formulations were placed in the outer tube of Centrisart set. The set was then placed in a cooling centrifuge (Sigma 3-30KS, Sigma Laborzentrifugen GmbH, Osterode, Germany) at 2000 rpm and 4 °C for 10 min. The supernatant was collected in the inner tube of Centrisart set since ultrafiltration occurs in the opposite direction of the centrifugal force. The amount of QCT in the supernatant was determined spectrophotometrically. For this purpose, serial dilutions of QCT solution in ethanol were measured at 375 nm using Cary 60 UV-Vis Spectrophotometer, Agilent, (Santa Clara, CA, USA) and a calibration curve was obtained. The method showed good linearity over the concentration range (0.004–0.1 mg/mL) with R^2^ = 0.999. The following equation was used to determine the EE:

(1) $$\mathrm{EE}\left(\%\right)=\left[\frac{\mathrm{Intial}\;\mathrm{QCT}\;\mathrm{concentration}\;-\mathrm{QCT}\;\mathrm{concentration}\;\mathrm{in}\;\mathrm{supernatant}}{\mathrm{Intial}\;\mathrm{QCT}\;\mathrm{concentration}}\right]\times 100$$

To calculate drug loading (DL), the quantity of QCT in a certain weight of lyophilized SLNs was determined.

(2) $$\mathrm{DL}\left(\%\right)=\left[\frac{\mathrm{Weight}\;\mathrm{of}\;\mathrm{QCT}\;\mathrm{in}\;\mathrm{SLNs}}{\;\mathrm{Weight}\;\mathrm{of}\;\mathrm{SLNs}}\right]\times 100$$

##### In Vitro Drug Release and Release Kinetics

Appropriate volumes of QCT-loaded SLNs (equivalent to 1 mg QCT) were placed in dialysis bags (Visking^®^, MWCO 12,000–14,000; SERVA, Heidelberg, Germany). The bags were placed in flasks containing 20 mL of release media (PBS with 0.7% Tween 80 to ensure sink conditions, pH 6.2 to mimic that of urine) in a shaking water bath (Wisebath^®^, London, UK) at 37 °C, 100 rpm. Samples of the release media were withdrawn at predetermined time intervals and the amount of QCT was determined spectrophotometrically at 375 nm.

After sample collection, the withdrawn medium was replaced with fresh buffer throughout the first 22 h of sampling. Afterwards, the release medium was entirely replaced each time with a new buffer, as previously reported by Patra et al. [39]. Dialyzed QCT from ethanol solutions was also quantified under the same conditions. To identify the release mechanism, data from release experiments were fitted to different release kinetic models (zero-order, first-order, Hixon Crowel, Higuchi, and Korsmeyer-Peppas). Experiments were done in triplicates.

##### Storage Stability 

Over the course of 3 months, SLNs dispersions were kept at 4 °C and their PS, PDI and %EE were analysed. The obtained results were compared to the zero-time data of the freshly prepared formulations.

##### Differential Scanning Calorimetry (DSC) 

For thermal analysis, differential scanning calorimeter (Perkin Elmer instruments, Model DSC 6, Los Angeles, CA, USA) was employed. Samples (5 mg) of QCT, pure lipids, SLNs and Cs were sealed in an aluminium pan and heated over a temperature range from 40 to 400 °C at a constant heating rate of 10 °C/min. Another empty aluminium pan was used as a reference.

##### Cell Viability Assay

1. Cell Culture

T-24: Urinary Bladder cancer cell line was obtained from Nawah Scientific Inc., (Cairo, Egypt), maintained in DMEM/RPMI-1640 containing 100 units/mL of penicillin, 100 mg/mL of streptomycin and 10% of heat-inactivated fetal bovine serum in humidified, 5% (*v*/*v*) CO_2_ atmosphere at 37 °C.

2. Cell Viability Assay

Sulforhodamine B (SRB) assay was carried out to assess cell viability [40]. In 96-well plates, 100 μL of cell suspension (5 × 10^3^ cells) were seeded and incubated in complete media for 24 h. Cells were then incubated with another media (100 μL) containing free QCT solution and QCT-loaded SLNs at various concentrations (equivalent to 0–90.6 μg/mL QCT) for another 72 h. For cell fixation. the media were replaced with of 10% trichloroacetic acid (150 μL) and incubated for 1 h, at 4 °C. After incubation, trichloroacetic acid was removed and cells were washed 5 times with distilled water. Seventy microlitres of SRB (0.4% *w*/*v*) were then added and incubated for 10 min in a dark place at 25 °C. After 3 washing cycles with 1% acetic acid, plates were left overnight to allow air-drying. In order to dissolve protein-bound SRB stain, 150 μL of Tris buffer (10 mM) were added. Finally, the absorbance was measured at 540 nm using a BMG LABTECH^®^-FLUO star Omega microplate reader (Ortenberg, Germany).

##### Cellular Drug Uptake and QCT Analysis Using High Performance Liquid Chromatography (HPLC)

Cells were seeded in T25 flask (Greiner bio-one, Frickenhausen, Germany) for 24 h. After reaching ~80–90% confluence, cellular uptake assay was performed. Cells were treated with fresh media (100 μL) containing the QCT-loaded SLNs (equivalent to 5 μg/mL QCT). Following incubation for 4 h, cells were washed two times and harvested using trypsin/EDTA. Then, pellets were collected by the cooling centrifuge. QCT was extracted by ethanol and quantified by HPLC. QCT was analysed by Waters (Milford, MA, USA) 2690 Alliance HPLC system equipped with reversed phase stainless steel column (250 mm × 4.6 mm), packed with 5 μm particles (Inertsil ODS-3V, Dikma Co., Foothill Ranch, CA, USA) and a Waters 996 photodiode array detector. The mobile phase was composed of 0.1% phosphoric acid in water and acetonitrile (60:40, *v*/*v*). The flow rate was adjusted to 1 mL/min, the wavelength of UV detector was kept at 254 nm while samples (10 μL) were injected into the column. All procedures were performed at room temperature. The method showed good linearity (R^2^ = 0.994) over the tested concentration range (50–250 ng/mL).

#### 2.2.2. Preparation and Characterization of Mucoadhesive In Situ Gel

In order to enhance their residence time inside the bladder and avoid their washout by periodic urination, optimized SLNs were planned to be incorporated into an intravesical thermosensitive mucoadhesive in situ gel system. Poloxamer 407 (P407) was employed as a thermosensitive in situ gel, together with mucoadhesive polymers such as Hydroxy propyl methyl cellulose (HPMC), Chitosan (Cs) and Carbopol 974P (Cb) and the obtained in situ gels were characterized.

##### Preparation of Plain and SLNs Loaded Mucoadhesive In Situ Gels 

For the development of P407 based in situ gels, the previously reported cold technique was adopted [41]. Specified amounts of poloxamers (P407/P188) were added to cold distilled water (4 °C) maintained in an ice bath under magnetic stirring. To get clear solutions, dispersions were kept overnight in the refrigerator [42]. Different poloxamer based in situ gels were prepared and characterized, and the optimized gel was then chosen for further experiments. For the preparation of mucoadhesive gels, calculated amounts of the mucoadhesive polymers (HPMC, Cb and Cs) were added to a certain volume of distilled water (or 1% acetic acid for Cs) under magnetic stirring at room temperature to obtain the desired concentrations of the mucoadhesive gels. The obtained mucoadhesive gels were left to cool to 4 °C in a refrigerator. Afterwards, the mucoadhesive gels were slowly dispersed in poloxamer solutions while being continuously stirred with a magnetic stirrer in an ice bath.

For preparation of SLNs loaded in situ gel, specified amount of P407 was well dissolved in SLNs suspension under magnetic stirring in an ice bath and was kept in a refrigerator overnight. The obtained dispersions were added to the previously prepared mucoadhesive gels following the same aforementioned technique. The effect of the incorporation of SLNs was investigated.

##### Characterization of Poloxamer Based In Situ Gels 

Gelation temperature was investigated following Donovan and Miller technique [43]. Ten millilitre flat-bottomed tubes containing 2 mL of the test formulations were placed in a water bath and heated to certain temperatures (18–32 °C). At each new setting, the tubes were allowed to equilibrate for 5 min and the formulations were assessed for gelation. The temperatures at which the formulations showed no flow and the meniscus failed to move, after the tubes were turned upside down, were recorded as the gelation temperatures.

Gelation time was investigated at the specified temperatures by the test tube inversion technique [44]. Formulations (2 mL) were maintained in a water bath at their predetermined gelation temperatures. Every few seconds, the tubes were removed and inverted to inspect on the state of the sample.

Erosion time was determined by placing 2 mL of the gel in a 10 mL flat-bottomed tube. The tube was left for equilibrium for 30 min at 37 °C to ensure gel formation. Two millilitres artificial urine (AU), pH 6.2 (details mentioned in subsequent section) were also equilibrated at 37 °C and cautiously placed on the gel surface. The tubes were kept in a water bath at 37 °C and visually inspected every 15 min until the gel was completely dissolved.

In vitro mucoadhesion of formulations was evaluated by following a reported agar plate method with little modification [45]. About 10 mL of agar 1%/mucin 2% *w*/*v* were placed in a 10 cm diameter-sterile Petri dish and left to cool. Two hundred milligrams of the in situ gels were placed at the center of the plate that was inclined by an angle of 60° for 15 h at 37 °C. The distance travelled by the sample on the plate was measured and it was inversely correlated to the adhesion potential of the gel.

Viscosity of the formulations (250 μL) was examined by Brookfield viscometer (Rotating viscometer, Brookfield DV II-RV, New York, NY, USA) fitted with S-52 spindle at 10 rpm. The temperatures were kept at 4 ± 1, 25 ± 1 and 37 ± 1 °C to measure the viscosities of the formulations.

Gel strength of different formulations was measured using a texture analyser (CT3; Brookfield, MA, USA) coupled with a 10,000 g load cell at 25 °C. A 36 mm diameter cylinder probe was pressed into beakers containing 10 gm of the formulations to a predetermined depth of 4 mm, at a rate of 0.2 mm/s. Hardness was determined using the obtained the force-time curves.

The percentage of in situ gel that can be pushed through the catheter was calculated using a technique reported by Chatta et al. with slight modification [46]. For this purpose, a urethral catheter (Well Lead Medical Co., Ltd., Guangzhou, China) of silicone coated tubing (outer diameter, 8 mm and length, 40 cm) was employed. Ten millilitres of in situ gel (at 4 °C) were weighed, placed into a 20-mL syringe and pushed through the catheter into a pre-weighed beaker. The collected gel was then weighed. To calculate the percentage of gel that was pushed through the catheter, this weight was divided by the initial gel weight and multiplied by 100.

In vitro drug release from in situ gel formulations was performed in order to compare release profile of QCT from SLNs loaded in situ gels with that from SLNs as such. Calculated amount of SLNs loaded in situ gels (equivalent to 1 mg QCT) in the sol phase at 4 °C were placed in dialysis bags. The bags were then kept in a water bath at 37 °C, which ensured gel formation. QCT release from SLNs loaded in situ gels was studied under the same conditions as that from SLNs alone.

#### 2.2.3. Ex Vivo Studies

##### Preparation of Artificial Urine

To prepare artificial urine (AU), a protocol reported by Mun et al. was followed [47]. The following ingredients were added to deionized water, using a magnetic stirrer at room temperature until completely dissolved, then the total volume was made to 2L: Na_2_HPO_4_ (0.11 g), NaH_2_PO_4_·H_2_O (1 g), Na_2_SO_4_ (0.26 g), NaHCO_3_ (0.34 g), MgSO_4_·7H_2_O (1 g), CaCl_2_ (0.67 g), NH_4_Cl (1.61 g), KCl (4.5 g), NaCl (6.34 g), and urea (24.27 g). The pH of the prepared artificial urine was 6.2. Throughout the ex vivo studies, artificial urine temperature was maintained at 37 °C.

##### Retention on Bladder Mucosa 

The retention capabilities of SLNs and SLNs loaded in situ gels on bovine bladder mucosa were examined following previously reported protocol with minor modifications [47]. Fluorescently labelled SLNs were used for the studies. Freshly extracted bovine urinary bladders were obtained from slaughter house in a cold box, stored at 0 °C, before being used for retention studies. Defrosted tissues were carefully cut into sections measuring about 2 × 2 cm^2^ without touching the internal mucosa, which were then used in the experiments. With their mucosal surface facing up, sections of the bladder tissues were mounted on a glass slide and washed with AU (5 mL). Background fluorescence microscopy images were taken initially for each tissue section using (Olympus BX-41, Tokyo, Japan). Then, 200 μL of different formulations were pipetted onto the bladder mucosa and images were again recorded. The bladder mucosa, being placed on a glass slide, were then washed 5 times with 10 mL of artificial urine at a rate of 2 mL/min. After each washing cycle, fluorescence microscopy images were taken again. Image J software was used to analyze the recorded microscopy images. Average fluorescence values following initial treatment and each washing cycle were calculated. The obtained values were used to produce a histogram, where the percentage retention is plotted against the volume of AU utilized. A 100% retention was set for the pre-wash fluorescence. Furthermore, Wash Out50 (WO_50_) values were used to quantify mucosal retention of the formulations. WO_50_ value denotes the volume of AU needed to remove 50% of the formulation from the urinary bladder mucosa [47]. Wash-out profiles were obtained by polynomial fitting and WO_50_ values of formulations were calculated by extrapolating to 50% retention. Experiments were done in triplicates.

##### Mucosal Penetration

Mucosal penetration studies were conducted as previously reported by Mansfield et al. [48]. Tissue sections of bladder mucosa (2 × 2 cm^2^) were treated with 200 μL of fluorescently labelled SLNs or SLNs loaded in situ gels. Samples were allowed to incubate for 4 h at 37 °C. At the end of the 4 h, bladder mucosa were washed with PBS and standard procedures were carried out to prepare 5 μm cross-sections using rotary microtome (CUT 5062, SLEE medical GmbH, Nieder-Olm, Germany). They were fixed on glass slides, scanned using the confocal laser scanning microscopy (CLSM) (Leica DMi8, Leica Microsystems, Wetzlar, Germany) and images were recorded using LAS X software. Deionised water was also used as a control. While obtaining fluorescent images, all samples were excited at 457 nm. Image J software was used to determine penetration into the bladder wall. Images were first converted to grayscale and intensity profiles of several lines were measured [49]. Experiments were done in triplicates.

##### Histopathological Evaluation 

Aliquots (200 μL) of QCT-loaded SLNs and SLNs loaded in situ gels were pipetted onto bladder mucosa (2 × 2 cm^2^). Samples were left to incubate for 4 h at 37 °C. Following incubation, bladder mucosa were washed to remove excess formulations and preserved in 4% neutral-buffered formalin. Five micrometer thick tissue sections were prepared following standard procedures, specimens were then stained by hematoxylin-eosin (HE) and histopathologically examined using a light microscope (Olympus BX-41, Tokyo, Japan). Deionised water was used as control. For each formulation, 6 different bladder tissue samples were examined to evaluate the tissue damage of healthy mucosa.

#### 2.2.4. Statistical Analysis

Data were compared for significance using Student’s *t*-test. Values less than 0.05 were considered statistically significant.

## 3. Results and Discussion

### 3.1. Preparation and In Vitro Characterization of SLNs

Various solid lipids were screened as a fundamental part of pre-formulation studies. According to previous literature, the use of lipids with high monoglyceride content produces less stable SLNs; thereby, lipids having no or low monoglyceride content were chosen [50]. QCT showed highest solubility in precirol and stearic acid, as demonstrated by the complete disappearance of drug crystals in the molten lipids. Precirol and stearic acid are generally recognized as safe (GRAS) and have been extensively employed as solid lipids for the development of SLNs [51,52]. Thus, for further studies, precirol was employed as an example of mixture of mono-, di- and triglycerides, and stearic acid was used as an example of fatty acids.

During SLNs fabrication, the particle size is reduced, which creates new surfaces, increases attraction forces between the nanoparticles, as well as surface tension at their interface, causing the system to become unstable. Thus, the addition of a stabilizer to the formulation is extremely important as it decreases surface tension and the surface energy of the system while imparting a repulsion force between particles [53]. Therefore, the proper choice of stabilizer type and concentration is crucial to ensure the fabrication of highly stable nanoparticles [54,55]. According to previous literature, different types of stabilizers having different molecular weight and charge have been successfully utilized to produce stable SLNs. In the current work, two non-ionic stabilizers (Tween 80 and P188) were screened and the proper one was selected for further investigations for the development of SLNs (Table 1).

#### 3.1.1. Effect of Formulation Variables on % EE and PS of SLNs

Particle size and entrapment efficiency of SLNs are greatly influenced by stabilizer composition and lipid to drug ratio [56]. As a part of formulation optimization studies, the influence of stabilizer type, concentration and lipid to drug ratio on entrapment efficiency and particle size of the formulated SLNs was investigated.

##### Effect of Stabilizer Type and Concentration on % EE and PS of SLNs

Tween 80 and P188 have been employed as stabilizers at various concentrations (1.25, 2.5, 5% for Tween 80 and 0.1, 0.5, 0.75% for P188). To examine the effect of stabilizer type on entrapment efficiency, as well as particle size of SLNs formulations, the medium concentration of both stabilizers was selected (2.5% Tween 80 and 0.5% P188) while keeping lipid concentration constant (Pr-QCT-SLNs-2, Pr-QCT-SLNs-9). Results in Table 1 show that P188 stabilized SLNs showed better QCT entrapment, while Tween 80 stabilized SLNs showed remarkably lower particle size.

For P188 stabilized formulations, %EE was about 98%. Such high %EE values can be corelated to P188 structure and its hydrophile–lipophile balance (HLB) value [57]. Also, P188 addition to the aqueous phase increases its viscosity, thereby reducing the diffusion of drug molecules to the aqueous phase and increasing their entrapment [58]. On the other hand, %EE for Tween 80 stabilized formulations was between 45 and 65%. Lower drug entrapment in the case of Tween 80 stabilized nanoparticles might be explained by the “partition phenomenon”. The presence of Tween 80 in the external phase increases drug solubilization in it, which promotes its diffusion from internal to external phase and decreases its entrapment inside SLNs [59]. This effect becomes more pronounced by increasing Tween 80 concentration. For example, SLNs stabilized with 1.25% Tween 80 showed an EE of 65% compared to 45.6% in the case of SLNs stabilized with 5% Tween 80.

A lower particle size of Tween 80 stabilized SLNs compared to P188 stabilized SLNs was also previously reported [60,61]. Tween 80 has an HLB value of 15, while the HLB value of P188 is 29. A smaller PS obtained in the case of Tween 80 stabilized nanoparticles could be attributed to its lower HLB value [60]. The particle size decreased proportionally with increasing stabilizer concentration, where at higher concentrations more stabilizer molecules were available to cover the interface, reducing the surface tension between the lipid and aqueous phases. Furthermore, particle coagulation was prevented by the steric barrier created by stabilizer molecules around the surface of nanoparticles [62]. For example, doubling Tween 80 concentration from 1.25% to 2.5% resulted in decreasing the particle size from 182 to 131 nm. Concerning P188 stabilized formulations, increasing P188 concentration from 0.1% to 0.5% (Pr-QCT-SLNs-4, Pr-QCT-SLNs-5) also resulted in lowering particle size. However, upon increasing P188 concentration above 0.5% (Pr-QCT-SLNs-6), the particle size increased, and this was also reported by Youssef et al. [43]. Excess stabilizer in solution can cause supersaturation of the stabilizer at the interface, induced by a diffusion process [63]. The presence of a high number of stabilizer molecules tends to increase particle size, which could be attributed to a higher degree of accumulation on the particle surface, as well as bridging effects, promoting aggregation.

The biological activity of QCT is dose and time dependent, as reported by many studies. Consequently, achieving high QCT entrapment and loading is essential and highly desirable [64]. Thus, P188 (0.5%) was selected for stabilization of SLNs, as it ensured fabrication of particles with an acceptable particle size, together with very high QCT entrapment.

##### Effect of Lipid to Drug Ratio on %EE and PS of SLNs

Lipid to drug ratio showed no effect on EE. SLNs prepared with different lipid to drug ratios using P188 as a stabilizer displayed excellent QCT entrapment (98–99%), which may be explained by its highly lipophilic nature [65].

Regarding the effect of lipid to drug ratio on PS, increasing lipid to drug ratio from 5:1 to 10:1 resulted in decreasing particles size from 346 to 220 nm for precirol based SLNs (Pr-QCT-SLNs-7, Pr-QCT-SLNs-8) and from 323 to 262 nm for stearic acid based SLNs (St-QCT-SLNs-1, St-QCT-SLNs-2) (Table 1). This decrease in PS can be explained by lower particle mobility due to higher viscosity of formulation at a higher lipid content, and hence particle aggregation is hindered [66]. Also, lower drug loading in case of lipid to drug ratio 10:1 led to the formation of more stable formulations which are less liable for aggregation. Bose et al. reported similar results, where upon increasing QCT loading into SLNs, the particle size increased [38]. However, further increasing the lipid to drug ratio from 10:1 to 15:1 resulted in increasing particle size from 220 to 304 in the case of precirol based SLNs (Pr-QCT-SLNs-8, Pr-QCT-SLNs-9) and from 262 nm to 359 nm for stearic acid based SLNs (St-QCT-SLNs-2, St-QCT-SLNs-3). The same results were previously reported [27,67]. Upon increasing the lipid concentration, the viscosity of the system increases, homogenization efficiency decreases and larger particles are produced [67]. Thus, lipid to drug (l0:1) was chosen as it yielded the smallest PS.

#### 3.1.2. Colloidal Characteristics, EE and DL of Selected Formulations

The particle size of selected formulations (Pr-QCT-SLNs-8 and St-QCT-SLNs-2) was 220 and 262, respectively. The PDI of the formulations was 0.32 and 0.23, respectively, demonstrating an acceptable particle size distribution and a low susceptibility for agglomeration [68].

Pr-QCT-SLNs-8 and St-QCT-SLNs-2 showed zeta potential values of −33 and −34 mV respectively. The obtained zeta potential values were close to previously reported QCT loaded SLNs compromising compritol and stearic acid [38,69]. Zeta potential determination is essential to anticipate the long-term stability of colloidal systems. In general, highly charged particles are less prone to aggregate during storage. Typically, a stable system has a zeta potential of ±20 mV or more [70].

Selected SLNs also ensured a high EE of QCT of about 98%, together with good drug loading up to nearly 8.8%. Interestingly, the maximum entrapment of QCT in Palmitic acid SLNs was 46.2% [71]. Another study of QCT delivery to the cornea and sclera via SLNs and nanoemulsion reported an EE of 66.5% and 74.2%, respectively [72]. QCT loading in this study was 8.87% compared to a maximum of 0.9% in the case of SLNs, nanostructured lipid carriers (NLCs) and lipid nanoemulsions [64]. Furthermore, Pinheiro et al. reported loading of only 10 mg QCT in 500 mg lipids for the preparation of SLNs and NLCs [73]. The high drug encapsulation provided by our study in comparison to the previously mentioned ones may be attributed to the proper selection of the surfactant type and concentration.

#### 3.1.3. Chitosan Coating

Chitosan (Cs), a natural cationic polysaccharide made up of d-glucosamine and N-acetyl glucosamine, has various advantages including biomembrane permeability, favourable muco-adhesiveness, and low toxicity [74]. Hence, the use of Cs together with SLNs may enhance drug permeation through mucosal barriers, prolong its residence in the target organ and accordingly improve its bioavailability [75]. Grabnar et al. examined the influence of Cs on urinary bladder wall in terms of permeation. It was found that urinary bladder wall permeability was increased significantly in the presence of Cs, supporting its use for the production of IDD systems [76].

To prepare chitosan coated SLNs, Cs solutions were added to the aqueous phase during SLNs preparation. Chitosan coating takes place due to electrostatic interaction between amino groups of chitosan and the lipid component of SLNs. In order to optimize PS and zeta potential of coated nanoparticles, the Cs solution of different concentrations was used, and the final pH was adjusted. A minimum concentration of 0.025% (pH = 5) and 0.1% (pH = 3.8) Cs solutions was adequate in achieving acceptable positive zeta potential and PS for precirol (C-Pr-QCT-SLNs-8) and stearic acid (C-St-QCT-SLNs-2) nanoparticles, respectively.

Coating SLNs with Cs was confirmed by a change in zeta potential values from negative to positive side (Table 1). Previous literature reported similar findings for various Cs-coated SLNs [77,78]. Noteworthily, Cs coating led to a remarkable increase in particle size from 220 to 243 nm in the case of C-Pr-QCT-SLNs-8, while following the coating of St-QCT-SLNs-2, a particle size increase from 262 to 275 nm was detected.

#### 3.1.4. Morphology of Plain, Coated and Uncoated SLNs

**Figure 1:**
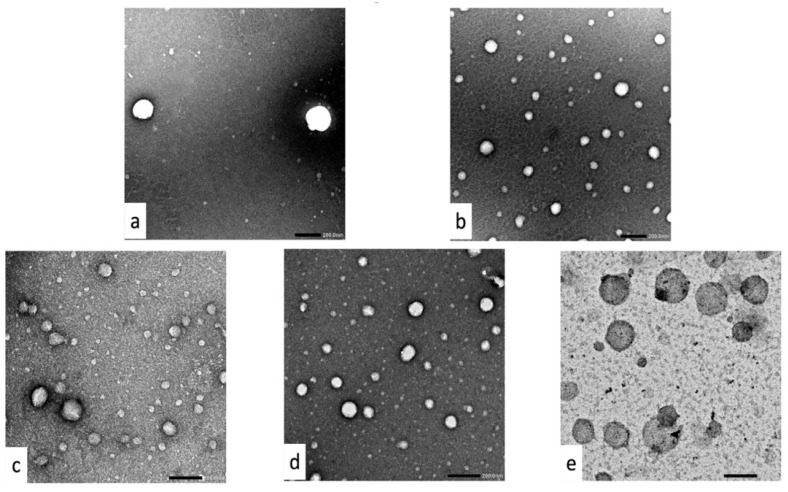
TEM images of different SLNs formulations: (**a**) Pr-SLNs-8; (**b**) Pr-QCT-SLNs-8; (**c**) St-SLNs-2; (**d**) St-QCT-SLNs-2; (**e**) C-St-QCT-SLNs-2. Abbreviations: QCT: quercetin, Pr: precirol, St: stearic acid, C: coated, SLNs: solid lipid nanoparticles.

TEM images of SLNs show small, sphere-shaped, homogenously distributed nanoparticles (Figure 1). Compared to SLNs with other shapes (disc-like, ellipsoidal or platelet-like), spherical particles possess smaller specific surface area with lower exposure to external medium; hence, they ensure higher drug encapsulation and more controlled release properties. Furthermore, stable spherical nanoparticles can be prepared using smaller concentrations of a stabilizer [79]. During sample preparation and prior to imaging, the hydration sheath surrounding the nanoparticles evaporates, which causes nanoparticles to appear with a smaller diameter than that obtained by the Zetasizer [80]. Cs coatings could be visually confirmed in TEM images with a relative increase in size. Also, C-St-QCT-SLNs-2 showed rough surface compared to the smooth one of St-QCT-SLNs-2. The absence of drug crystals in TEM images of coated and uncoated QCT-loaded SLNs indicates high QCT entrapment inside the nanoparticles.

#### 3.1.5. In Vitro Release of QCT from Nanoparticles

**Figure 2:**
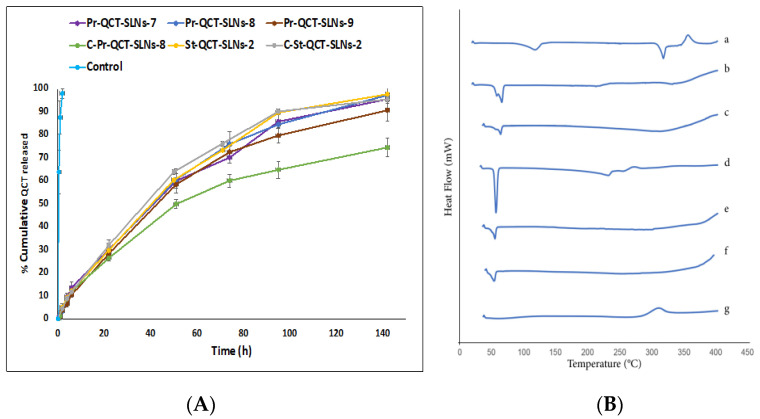
(**A**) In vitro QCT release from different SLNs formulations in PBS supplemented with 0.7% Tween 80, pH = 6.2 at 37 °C. (**B**) DSC thermogram of different SLNs and their components (**a**) QCT, (**b**) Precirol, (**c**) Pr-QCT-SLNs-8, (**d**) Stearic acid, (**e**) St-QCT-SLNs-2, (**f**) C-St-QCT-SLNs-2, (**g**) Chitosan. Abbreviations: QCT: quercetin, Pr: precirol, St: stearic acid, C: coated, SLNs: solid lipid nanoparticles. The numbers 7,8 and 9 donate lipid to drug ratios 5:1, 10:1 and 15:1 respectively.

A QCT solution in ethanol was completely dialysed after 2 h (Figure 2A). However, the QCT release from SLNs displayed a biphasic pattern with relatively constant slow-release phase over the first 95 h and then a very slow-release phase up to 142 h. The percentage of QCT released from different SLNs in the first 22 h was about 30%. Meanwhile, QCT was completely released from the SLNs in about 142 h. There was no statistically significant difference between percentage of QCT released from precirol based SLNs containing different lipid to drug ratios or even between precirol and stearic acid based SLNs with the same lipid to drug ratios (Pr-QCT-SLNs-8, St-QCT-SLNs-2) at 22, 95 and 142 h, which may be explained by the high affinity of QCT to both precirol and stearic acid, as also demonstrated by high %EE values. Similar results were described by Hazzah et al., where SLNs with different lipid types stabilized by Gelucire50/13 (8%) showed a similar release profile [81].

For precirol based formulations, Cs coating further impeded release profile compared to uncoated SLNs. The presence of Cs layer around SLNs increases the diffusional distance for QCT to be released into the release medium [82]. Therefore, both the extent and the rate of QCT release were lowered after Cs coating and more of a controlled release profile was attained in the case of C-Pr-QCT-SLNs-8. On the other hand, the stearic acid-based SLNs release profile showed no significant difference between coated and uncoated formulations. One possible explanation is that there was no great difference between the particle sizes of St-QCT-SLNs-2 and C-St-QCT-SLNs-2. Hence, they exhibited similar release profiles.

SLNs are adequate in achieving a prolonged drug release. The controlled release properties provided by SLNs can be attributed to the strong hydrophobic interaction between lipids compromising SLNs and the drug, in addition to the ability of SLNs to retain their solid state at body temperature [82]. Furthermore, a QCT which is highly lipophilic molecule could be solubilized in the molten lipids and crystallize inside the core, giving rise to drug-enriched core model SLNs wherein a layer of drug-free lipids surrounds the lipid core in which the drug is concentrated. This layer increases the diffusion path length and retard drug release, providing a sustained release profile [83]. Thus, encapsulating QCT in SLNs ensured its sustained delivery over a prolonged period, making them excellent candidates for IDD. To avoid or minimize the burst effect, SLNs were produced by using a stabilizer that is not able to solubilize the drug [84]. Our release profile was relatively similar to that from polymeric micelles reported by Patra et al. using a similar medium [39]. These micelles were also composed of a hydrophobic central core of polypropylene oxide, ensuring high QCT encapsulation inside the nanoparticle, surrounded by a hydrophilic shell of polyethylene oxide protecting the drug from the external environment. In comparison with prolonged QCT release up to 142 h provided by our SLNs, SLNs prepared by other lipids, stabilizers and methods showed almost complete QCT release from SLNs within 12–48 h [85,86]. After fitting data obtained from release studies to several kinetic models, the obtained regression values (r^2^) (Table 2) revealed that QCT release from SLNs followed the Korsmeyer Peppas model. In addition, the release of QCT was governed by the “Non-fickian” mechanism, as the diffusional exponent (n) values were between 0.6 and 0.8. It describes a condition where the formulations follow the hybrid of diffusion and erosion mechanism. A similar trend was reported for indomethacin release from SLNs [87]. As C-Pr-QCT-SLNs-2 exhibited remarkably lower release profile compared to other SLNs, they were excluded from further studies.

#### 3.1.6. Stability Study

Stability studies revealed relative stability of SLNs, where a slight particle size increase of 35 and 36 nm was observed after three months in the case of St-QCT-SLNs-2 and C-St-QCT-SLNs-2, respectively (Table 3). Although the difference in PS of these formulations after three months storage with respect to the freshly prepared samples was statistically significant, particle size remained in the acceptable range after the whole storage period. The simple visual inspection did not show any drug precipitation during storage.

For all formulations, PDI values of freshly prepared formulations and after three months storage were less than 0.4. This is suggestive for a homogenous system with an acceptable particle size distribution. Also, there was no change observed in % EE beyond the three months.

#### 3.1.7. Thermal Properties of SLNs Using DSC 

DSC is a useful analytical technique to investigate the recrystallization and melting behaviour of various materials. QCT, precirol, stearic acid, chitosan, as well as different SLNs were analysed using DSC. As Figure 2B illustrates, QCT thermogram displayed two endothermic peaks. The first peak is observed at 117.29 °C and correlates to a decomposition process, where water is released from the crystal lattice. Because of the strong hydrogen bonding between QCT and water, the peak appeared at high temperature (117.29 °C). The second peak appeared at 317.6 °C and is correlated to QCT melting temperature. A QCT thermogram also showed an exothermic peak at 356.16 °C corresponding to its thermal degradation [88]. Precirol displayed an endothermic peak at 64 °C, denoting its melting point; the presence of a small shoulder beside the main peak might indicate the presence of other polymorphs [29]. On the other hand, stearic acid exhibited a sharp melting peak at 56 °C. Compared to bulk lipids, SLNs showed melting transition at slightly lower temperatures, which could be attributed to the presence of a stabilizer around SLNs and the incorporation of drug molecules [89]. In the case of C-St-QCT-SLNs-2 where QCT and Cs were present together, the decrease in the melting transition was more pronounced, indicating the formation of less ordered crystals with a lower, tightly packed arrangement of lipids [78]. Moreover, SLNs showed lower enthalpy compared to pure lipids (Table 4), suggesting a disturbance of the crystalline structures and the formation of amorphous structures where for crystalline materials, more energy is required to overcome lattice forces [90]. Cs thermogram exhibited an exothermic peak which corresponds to its thermal decomposition (degradation of acetyled and deacetylated units, cleavage of glycoside bond and monomer dehydration) [91]. All SLNs showed melting temperatures over 40 °C, which proves that they would be able to retain their solid status at room, as well as body temperature. Also, the absence of QCT peak in thermograms of SLNs suggests good QCT encapsulation within the lipid matrix of SLNs.

#### 3.1.8. Cytotoxicity Assay

Previous studies showed that QCT has anticancer activity on various cancer cell lines [92]. Induction of apoptosis and cell cycle arrest were proposed as possible mechanisms for the anti-proliferative properties of QCT [93]. Although the anti-cancer effects of QCT on lung, breast, ovarian and colon cancer cells have been thoroughly studied [94], few research reports on QCT activity against bladder cancer. One aim of this research is to highlight QCT activity against bladder cancer.

**Figure 3:**
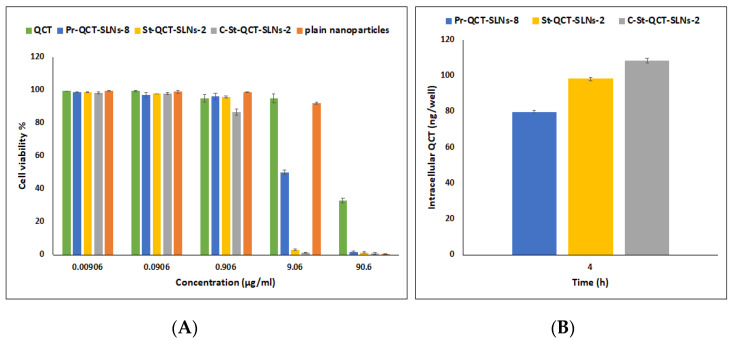
(**A**) A cytotoxicity profile of QCT, plain and QCT loaded SLNs formulations incubated with T-24 cells at different concentrations. (**B**) Cellular uptake of QCT from different SLNs at 4 h. Abbreviations: QCT: quercetin, Pr: precirol, St: stearic acid, C: coated, SLNs: solid lipid nanoparticles.

The cytotoxic effects of QCT solution, plain and QCT-loaded SLNs on T-24 cancer cell lines were investigated. Cells were incubated with QCT and SLNs (equivalent to 0–90.6 μg/mL QCT) for 72 h at 37 °C. The formulations showed a dose-dependent decrease in T-24 cell viability (Figure 3A). The IC_50_ values of free QCT solution, Pr-QCT-SLNs-8, St-QCT-SLNs-2 and C-St-QCT-SLNs-2 were found to be 57.68, 8.9, 2.5 and 1.66 μg/mL, respectively. A higher cytotoxic effect of QCT-loaded SLNs compared to free QCT can be explained by improved cellular uptake. Free QCT may be susceptible to efflux via p-glycoprotein pumps, while on the other hand, SLNs are internalized inside the cells by endocytosis and are not vulnerable to efflux via p-glycoprotein pumps [39]. This allows the drug to be retained inside the cells for prolonged time span and enhances its cytotoxic effects [39]. Previous literature also reported some inhibitory effects of P188 on p-glycoprotein pumps [95]. Furthermore, the slow drug release from SLNs may be also responsible for its higher cytotoxic effects [96]. The higher cytotoxic effects of drug loaded nanoparticles compared to free drug on different cell lines such as A549, MiaPaCa-2 and PPCL-46 was also reported by earlier literature [97,98]. Compared to Pr-QCT-SLNs-8, St-QCT-SLNs-2 showed significantly higher cytotoxicity. These results can indicate that cytotoxicity of SLNs can be influenced by the lipid matrix. Previous literature showed that free stearic acid based SLNs exerted higher cytotoxic effects compared to SLNs consisting of triglycerides of stearic acid [99]. Also, the higher cytotoxicity of stearic acid based SLNs might be explained by their enhanced cellular internalization [100].

Depending on their molecular weight, degree of deacetylation, dose and time of incubation, Cs solutions may show some degree of toxicity [78,101]. In the current study, Cs was used for coating SLNs in concentrations less than 1 mg/mL, and at these concentrations no relevant toxicity has been observed on respiratory epithelial cell lines when Cs is employed as a coating material [78]. Cs coated SLNs showed significantly (*p* < 0.05) decreased cell viability relative to uncoated SLNs, which was also consistent with the results obtained from the cellular uptake assay. Appling Cs as a coating material for SLNs allows for a lengthier contact time for the drugs to be transported across cellular membranes, enhancing their cellular uptake and accordingly their cytotoxicity [102]. Additionally, the structural similarity between Cs and hyaluronic acid, a naturally occurring ligand for CD44 receptor that is over expressed on membranes of bladder cancer cells, may explain its role in tumour targeting [103]. Plain SLNs did not show a considerable toxic effect on cell lines in concentrations up to 9.06 μg/mL; however, they displayed cytotoxicity at higher concentration equivalent to 90.6 μg/mL. Previous literature also reported some cytotoxic effects for plain SLNs [29,104]. Thus, encapsulating QCT in SLNs resulted in a high increase in its cytotoxic activity against T-24 cell lines, as demonstrated by low IC_50_ values compared to free QCT. In comparison to the very low IC_50_ obtained by our study, a previous study showed that following incubation of T-24 cells with QCT-coated titanate nanotubes for 48 h, a highly cytotoxic effect on cells was observed at 200 μg/mL [105].

#### 3.1.9. Cellular Drug Uptake

It was shown that the cellular internalization of drug-loaded nanoparticles and their sustained retention inside the cells have a great influence on their therapeutic effects [106]. All SLNs showed considerable uptake by T-24 cells (Figure 3B), where 80, 98 and 108 ng QCT were taken up by the cells for Pr-QCT-SLNs-8, St-QCT-SLNs-2 and C-St-QCT-SLNs-2, respectively, which represents about 16, 20 and 22% of the total added drug. Cs coated nanoparticles demonstrated the highest cellular uptake. The pH of tumour environment is acidic, which assures ionization of Cs chains. Cationic amino groups on chitosan molecules are able to interact with anionic phospholipid groups on cell membranes. Hence, coating SLNs with Cs promotes the electrostatic interaction between SLNs and cellular surfaces and boosts cellular uptake [103].

Previous literature demonstrated that the incubation of cells with QCT resulted in no uptake of molecule by cells due to its lower solubility properties [107]. On the other hand, findings obtained from our cellular uptake studies suggested that SLNs enhanced the uptake of QCT. Stearic acid based SLNs showed better cytotoxicity and cellular uptake in T-24 cell lines. Hence, they were chosen for further investigations.

### 3.2. Characterization and Optimization of the Mucoadhesive In Situ Gel Formulations 

An intravesical drug delivery system was developed by incorporation of the optimized formulations (St-QCT-SLNs-2 and C-St-QCT-SLNs-2) into a mucoadhesive, thermosensitive in situ gel. One of the most popular amphiphilic block copolymers with thermo-reversible properties in aqueous conditions is the poloxamer. At elevated temperatures, poloxamers change from a “zigzag” configuration into a viscous gel with closely-packed arrangement [35]. Because of their great drug release characteristics, low toxicity and high solubilizing power, poloxamers are often employed as a mucosal drug delivery platform [35].

To ensure easy intravesical application of the in situ gel and enhance its residence time in the urinary bladder for sustained drug release from the gel even after voiding, two main indices were evaluated; namely, gelation temperature and erosion time. Gelation temperature should ensure that the formulations form gels at body temperature while remaining in the liquid state at lower temperatures. The erosion time should be as lengthy as possible.

Preliminary studies were performed to investigate the gelation temperatures and erosion time of different concentrations of P407 alone and in combination with P188 (Table 5).

The gelation temperature of P407 (20%*w*/*w*) was 25 °C, as also demonstrated by previous studies [31,108], which means that it remains liquid at 4 °C and gels at 37 °C (body temperature). P407 (20%*w*/*w*) also showed a considerably high erosion time of up to 20.5 h. Hence, it was selected for further investigations as it yielded a gel with suitable gelation temperature for intravesical administration and a considerably lengthy erosion time.

As P407 lacks mucoadhesive properties, the incorporation of mucoadhesive polymers into the gel will ensure the development of mucoadhesive gels [109]. In the current work, HPMC, Cb and Cs were used in combination with P407 (20%*w*/*w*) to optimize muco-adhesion, gelation temperature and erosion time. These in situ gels were developed using materials that do not hinder the flow of urine, as they gradually become dissolved in urine, while remaining mucoadhesive for long duration to ensure drug uptake into the urothelium [5].

#### 3.2.1. Gelation Temperature

P407 undergoes sol-gel transition in response to temperature increase, which is explained by the decreased solubility of the polypropylene oxide (PPO) block of P407 at higher temperatures [110]. As shown in Table 5, gelation temperatures of P407 decreased at higher concentrations (G1–G5). A similar trend was observed in previous literature [111]. At high P407 concentration, above the micellar concentration, poloxamer unimers are associated into micelles, and when these micelles aggregate, a gel state is formed [110]. The addition of P188 to P407 resulted in an increase in gelation temperature, and this effect became more pronounced with increasing P188 concentration. Similar results were previously reported [112,113]. The molar ratio between propylene oxide (the hydrophobic block of poloxamers) and ethylene oxide (the hydrophilic block of poloxamers) is 0.28 and 0.17 for P407 and P188, respectively. By addition of P188 to the in situ hydrogel, the propylene oxide/ethylene oxide, this molar ratio is lowered, resulting in increased hydrophilicity and higher gelation temperatures [114].

On the other side, for Cb (1.0%) incorporated P407 gel, the gelation temperature (24.7 °C) was slightly lower than that of P407 gel formulation (25 °C), showing good agreement with the previous report [115]. Also, upon Cs (1.0%) addition, a decrease in the gelation temperature from 25 to 23.5 °C was observed; hence, Cs slightly affected the gelation temperature of P407 as also previously reported [109]. Meanwhile, in the case of HPMC incorporated P407 gel, the gelation temperature was decreased by 5 °C [116]. Mucoadhesive polymers are able to bind to the polyethylene oxide block of P407, promote dehydration and enhance the entanglement between adjacent molecules, which encourage gel formation at lower temperatures [117]. Finally, the incorporation of St-QCT-SLNs-2 into G9 showed no effect on gelation temperature of the mucoadhesive in situ gel, while C-St-QCT-SLNs-2 addition led to slight decrease in gelation temperature of G10 from 23.5 to 23 °C.

#### 3.2.2. Gelation Time

Another considerable parameter is gelation time. This is the time required for an in situ gel forming system to change from the liquid state to a gel state. P407 (20%) formulation demonstrated very short gelation time of about 11 secs (Table 5). Gelation time did not change significantly either after the addition of mucoadhesive polymers (Cb or Cs) or incorporation of SLNs into the system [118,119]. Such a short gelation time ensures fast gelation of the formulations in the target site, which protects them from being washed out shortly after administration [120].

#### 3.2.3. Erosion Time

The concentration of P407 is directly proportional to erosion time because the higher concentration of P407 results in greater gel strength (Table 5), and this is similar to another study [121]. Meanwhile, P188 (2.5, 5%) addition to P407 (20%) led to erosion time reduction from 20.5 to 18 and 15 h, respectively, where the high hydrophilicity of P188 favoured the erosion of the hydrogel in aqueous medium [114]. On the other hand, the incorporation of mucoadhesive polymers (both Cb and Cs) into the P407 based in situ gels resulted in erosion time prolongation. The incorporation of such polymers into P407 based gels leads to hydrogen bonds formation between P407 and these polymers, which augments the strength of the in situ gels and extends their erosion time [122]. For P407 based gels with Cb and Cs as mucoadhesive polymers, the erosion time was around 23 h, which is long enough to ensure high residence time and continuous uptake of SLNs by urothelial cells. Incorporation of SLNs into these gels further decreased their erosion rate, which was also previously reported by Dorraj et al. [118]. Based on previous results, both Cb (1%) and Cs (1%) with P407 (20%*w*/*w*) yielded gel with appropriate gelation temperature, gelation time and a considerably lengthy erosion time. Hence, gel with anionic polymer (Cb) was selected for incorporation of negatively charged SLNs (St-QCT-SLNs-2), while that with cationic polymer (Cs) was selected for incorporation of the positively charged SLNs (C-St-QCT-SLNs-2). The optimized in situ gel formulations were further characterized in terms of muco-adhesion, rheological, mechanical properties and their effect on drug release.

#### 3.2.4. Muco-Adhesion

When placing the optimized formulations on the top of a plate containing agar and mucin, they showed very slight displacement (5 mm) over 15 h, indicating good adhesion characteristics; hence, the mucoadhesion was recorded as “Good” (Table 5). The presence of large number of carboxylic groups in Cb structure enables it to form strong hydrogen bonds with the carboxylic groups located on the carbohydrate chain of mucin, and thereby a strengthened network between Cb and mucin is formed, promoting its mucoadhesion [123]. Cs is a well-known cationic polymer with a high adhesion potential to negatively charged surfaces [124]. The mucoadhesive properties of Cs can be correlated to both the hydrophobic interactions between the methyl groups of acetylated Cs chains and the methyl groups of mucins, as well as the electrostatic interactions of the cationic amino groups of Cs with the anionic sialic acid residues of mucins [125].

#### 3.2.5. Rheological and Mechanical Characteristics

**Figure 4:**
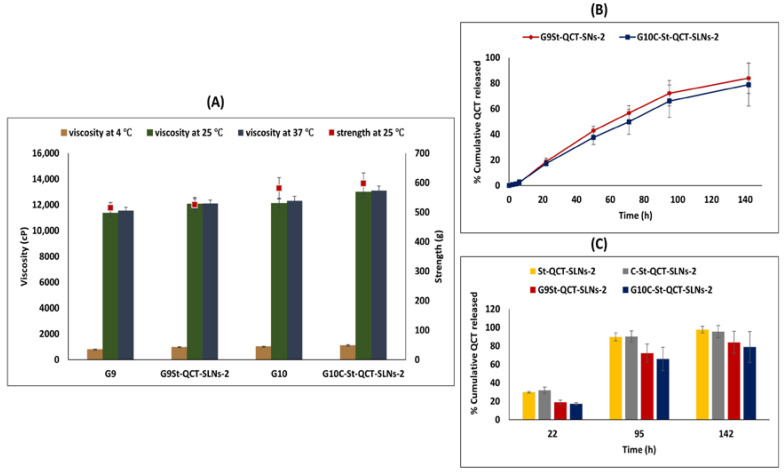
(**A**) The viscosity of selected in situ gel formulations at 4, 25 and 37 °C and their strength at 25 °C. (**B**) In vitro QCT release from P407 based gels and (**C**) Cumulative percentage of QCT released from different formulations at 22, 95 and 142 h, in PBS supplemented with 0.7% Tween 80, pH = 6.2 at 37 °C. Abbreviations: G_9_: carbapol/poloxamer 407 gel, G_10_: chitosan/poloxamer 407 gel, QCT: quercetin, St: stearic acid, C: coated, SLNs: solid lipid nanoparticles.

Previous literature reported a strong relationship between the rheological and mechanical characteristics of P407 based in situ gels, where low viscosity and weak mechanical strength can lead to the rapid erosion of P407 gels and affect their retention inside the bladder [126]. Meanwhile, very high viscosity and mechanical strength can cause problems during administration and patient inconvenience. Hence, the mechanical and rheological characteristics should be optimized to ensure a balance between long residence time of the formulations inside the bladder and easy administration. As Figure 4A illustrates, the viscosity of gels significantly increased (*p* < 0.05) from 966 and 1100 cP at 4 °C to 12066 and 12983 cP at 25 °C for G_9_St-QCT-SLNs-2 and G_10_C-St-QCT-SLNs-2, respectively, which is explained by the thermogelling property of P407 molecules at 25 °C. These findings show good agreement with tube inversion test results. However, when the temperature was increased from 25 °C to 37 °C, no significant increase (*p* > 0.05) in viscosity was observed as the formulations existed in gel state at both temperatures. Similar results were previously reported [118]. The use of Cs and Cb as mucoadhesive polymers yielded a gel with comparable viscosity at 25 °C. Similarly, gel loading with SLNs did not affect its viscosity at 25 and 37 °C. For all formulations, gel strength was between 515–597 g. Using Cs as a mucoadhesive polymer yielded gel with slightly higher strength than that obtained by using Cb. Finally, SLNs incorporation into gel led to an insignificant increase in its strength.

#### 3.2.6. Determination of the Percentage of Gels That Can Be Pushed through a Catheter

In order to ensure proper dosing of intravesically administered drugs dispersed in gels, sufficient volume of the gel should reach the bladder after injection into the catheter. Optimized in situ gel formulations were manually injected into catheters at 4 °C. Results shown in Table 5 demonstrated that at least 95% of the developed gels could be successfully and easily injected through the catheters, which can be explained by low gel viscosity at 4 °C.

#### 3.2.7. In Vitro Release of QCT from Gel Formulations

SLNs loaded in situ gels (G_9_St-QCT-SLNs-2 and G_10_C-St-QCT-SLNs-2) allowed sustained QCT release; where around 18% QCT was released during 22 h, reaching 80% after 142 h (Figure 4B). The release profiles of SLNs loaded P407 based gels was compared to that of SLNs (St-QCT-SLNs-2 and C-St-QCT-SLNs-2). The incorporation of SLNs into P407 based gel formulations led to significant retardation (*p* < 0.05) in QCT release; where about 18, 70% was released from SLNs loaded gel formulations compared to approximately 30, 90% from SLNs alone at 22 and 95 h, respectively (Figure 4C). Release rate from in situ gel system increased gradually afterwards; at 142 h, no significant difference (*p* > 0.05) in percentage of QCT released from in situ gel and SLNs was observed. Because of the reduced dimensions and number of water channels in its micellar structures, P407 is reported to retard the drug release rate [127]. The shorter the distance between adjacent micelles, the higher the number of cross-links between them and the lower the rate of drug release is. Furthermore, lipophilic drugs like QCT undergo slow diffusion from the in situ gel matrices as they have higher affinity toward the core of P407 micelle [128]. Another mechanism for the release of lipophilic drugs such as QCT is gel erosion, which occurs due to the dissociation of the micellar packing structure of P407 in the presence of aqueous media, leading to the degradation of the gel matrix and drug release [128]. Noteworthily, the gradual increase in QCT release rate from P407 based gels compared to SLNs may be directly correlated to gel erosion with time. Finally, QCT release from G_10_C-St-QCT-SLNs-2 was slightly lower than that from G_9_St-QCT-SLNs-2. This may be explained by the former having higher strength and a lower erosion rate [129]. After the release data was fitted to various models, it was found that QCT release from the in situ gel followed the Korsmeyer–Peppas model with r^2^ equivalent to 0.99. Furthermore, the n value obtained from the Korsmeyer–Peppas plots was around 0.98, indicating a non-Fickian release mechanism; thus, it projected that the in situ gel delivers QCT by coupled diffusion and erosion [130].

### 3.3. Ex Vivo Studies

#### 3.3.1. Retention of Selected Formulations on Mucosal Surfaces of Bovine Urinary Bladder

**Figure 5:**
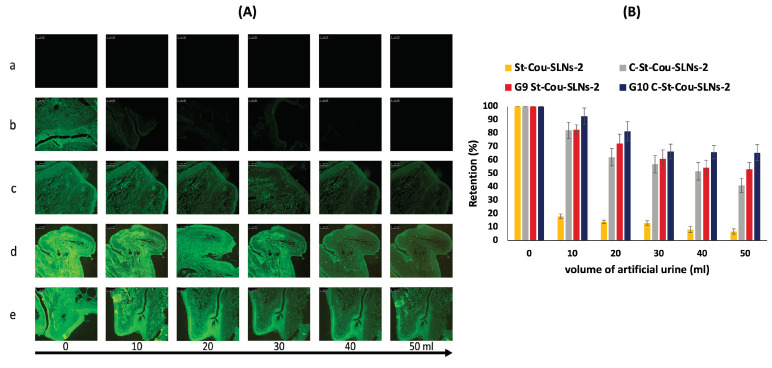
(**A**) Fluorescence images showing the retention of different formulations on bovine urinary bladder mucosa washed with different volumes of AU. (**a**) Control, (**b**) St-Cou-SLNs-2, (**c**) C-St-Cou-SLNs-2, (**d**) G_9_St-Cou-SLNs-2, (**e**) G_10_C-St-Cou-SLNs-2. (**B**) Percentage retention of St-Cou-SLNs-2, C-St-Cou-SLNs-2, G_9_St-Cou-SLNs-2 and G_10_C-St-Cou-SLNs-2 on bovine urinary bladder mucosa after washing with different volumes of AU. Abbreviations: G_9_: carbapol/poloxamer 407 gel, G_10_: chitosan/poloxamer 407 gel, Cou: coumarin-6 dye, St: stearic acid, C: coated, SLNs: solid lipid nanoparticles.

Since the developed formulations were intended for intravesical drug delivery, their mucoadhesive properties are important to ensure the retention of SLNs inside the bladder for prolonged time, allowing sufficient contact with the tumour cells. The retention properties of different formulations on bovine urinary bladder mucosa were assessed using fluorescent detection methodologies described in previous literature [47,131] with some modifications. The formulations were washed with artificial urine solutions for successive times and the amount of SLNs retained on the bladder mucosa was quantified. The experiment simulates the in-vivo conditions, where urine flow can affect the retention of SLNs inside the urinary bladder. For this purpose, fluorescently labelled SLNs were prepared using coumarin-6 (cou) instead of QCT. Figure 5A shows exemplary fluorescence images of untreated bladder mucosa (control) as well as bladder mucosa treated with different formulations (St-Cou-SLNs-2, C-St-Cou-SLNs-2, G_9_St-Cou-SLNs-2 and G_10_C-St-Cou-SLNs-2) and washed with different volumes of AU. Image J software was employed to quantify the retention of formulations on bladder mucosa.

St-Cou-SLNs-2 exhibited poor mucoadhesive properties; where only about 7% of the formulation was retained on bladder mucosa following 5 washing cycles with a total AU volume of 50 mL (Figure 5B). However, it was found that 41% of C-St-Cou-SLNs-2 was retained on the bladder mucosa after the same number of washing cycles confirming their very good mucoadhesive properties. On the other hand, gel loaded with SLNs showed the best retention on bladder mucosa where 53, 65.5% of formulations were retained on bladder mucosa until the end of washing cycles for G_9_St-Cou-SLNs-2 and G_10_C-St-Cou-SLNs-2, respectively. These results confirmed the mucoadhesive properties of Cs coated SLNs, which could be attributed to Cs nature. Being positively charged, Cs macromolecules can electrostatically interact with negatively charged sialic acid residues and other components of mucus rendering Cs mucoadhesive. Hydrophobic interaction and hydrogen bonding may also be responsible for the mucoadhesive properties of Cs [132,133]. Taking the advantage of their mucoadhesive property, in situ gel formulations loaded with SLNs were retained on the bladder mucosa after the five washing cycles. Similar results were demonstrated by Men et al., where incorporation of nanoparticles in P407 gel led to their in vivo retention on bladder wall of mice even after urination [31]. The higher retention properties demonstrated by G_10_C-St-Cou-SLNs-2 in comparison to G_9_St-Cou-SLNs-2 may be explained by the greater mucoadhesive effect provided by both Cs present in SLNs coating and gel. Interestingly, the percentage retained of G_10_C-St-Cou-SLNs-2 remained nearly the same for the last three washing cycles, which is a predictor of high retention properties even after washing by higher quantities of AU. By comparing WO_50_ values of different tested formulations, it can be concluded that both C-St-Cou-SLNs-2 and G_9_St-Cou-SLNs-2 (WO_50_ = 41.2 and 51.9 mL respectively) showed better retention on the bladder mucosa compared to St-Cou-SLNs-2 (WO_50_ = 5 mL), while the best retention was observed for G_10_C-St-Cou-SLNs-2 with WO_50_ value of 71.9 mL. Compared to previous literature reporting the use of different mucoadhesive systems for IDD [47,134,135], the prepared in situ hydrogel incorporating cationic SLNs displayed superior retention properties.

#### 3.3.2. Mucosal Penetration

**Figure 6:**
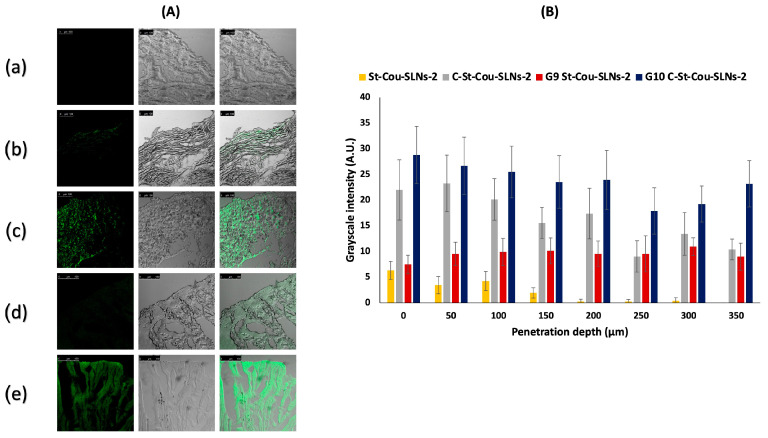
(**A**) CLSM images of cross-sections of bovine urinary bladder mucosa (**a**) Control, (**b**) St-Cou-SLNs-2, (**c**) C-St-Cou-SLNs-2, (**d**) G_9_St-Cou-SLNs-2, (**e**) G_10_C-St-Cou-SLNs-2. (**B**) Depth profile of fluorescence intensities. Abbreviations: G_9_: carbapol/poloxamer 407 gel, G_10_: chitosan/poloxamer 407 gel, Cou: coumarin-6 dye, St: stearic acid, C: coated, SLNs: solid lipid nanoparticles.

In order to investigate the penetration capabilities of St-Cou-SLNs-2, C-St-Cou-SLNs-2, G_9_St-Cou-SLNs-2 and G_10_C-St-Cou-SLNs-2 into the bladder mucosa, CLSM was employed. The CLSM technique is a useful imaging tool for the better understanding of penetration abilities of the nanocarriers into tissue layers [136]. CLSM of sections of the bladder wall (Figure 6A) shows control bladder with negligible fluorescence. For St-Cou-SLNs-2 treated bladder mucosa, fluorescence was confined to the upper layers of the bladder wall up to 150 μm (Figure 6B). Although G_9_St-Cou-SLNs-2 treated bladder mucosa showed nearly the same degree of fluorescence as St-Cou-SLNs-2 in the upper most layer, the fluorescence intensity remained the same up to 350 μm. On the other hand, Cs coated formulations showed significantly higher penetration abilities compared to uncoated ones. For instance, C-St-Cou-SLNs-2 showed remarkably higher fluorescence intensity at different depths compared to St-Cou-SLNs-2. The fluorescence intensity, however, decreased in deeper layers. Meanwhile, G_10_C-St-Cou-SLNs-2 showed the highest fluorescence in the upper as well as deeper layers of the bladder wall up to 350 μm. Chitosan as a cationic polymer may enhance drug ability to penetrate into deeper layers of the bladder wall. The previously mentioned electrostatic interaction between the polymer and the mucus layer of the bladder is believed to encourage the paracellular transport of drugs through the rearrangement of the tight junctions between urothelial cells [32]. Similarly, the mucoadhesive in situ gels were able to enhance fluorescently labelled SLNs penetration into the bladder wall, which can be directly correlated to their prolonged adherence to the urinary bladder mucosa in comparison with SLNs dispersions, as also proved by a retention study [49].

According to the anatomy of the bladder, the urothelium has a thickness of about 200 μm, while the lamina propria extends from 200 to 1200 μm [49]. Uncoated nanoparticles, St-Cou-SLNs-2, demonstrated good deposition on the urothelium surface with minimal penetration into deeper layers of the bladder, chitosan coated SLNs, C-St-Cou-SLNs-2, demonstrated high penetration into the upper layers of the bladder wall and lower degree of penetration into the lamina propria. Gel loaded Cs coated SLNs, G_10_C-St-Cou-SLNs-2, demonstrated very good accumulation on the upper urothelial layers of the bladder wall, together with high penetration into deeper layers of the lamina propria. This suggests that a particular modification could be considered for this SLNs loaded in situ gel, since it may be employed either as an IDD system for management of non myoinvasive bladder cancer or as a depot for the sustained delivery of drug-loaded SLNs into deeper layers of the bladder, allowing for greater control of myoinvasive bladder cancer.

#### 3.3.3. Histopathological Evaluation

**Figure 7:**
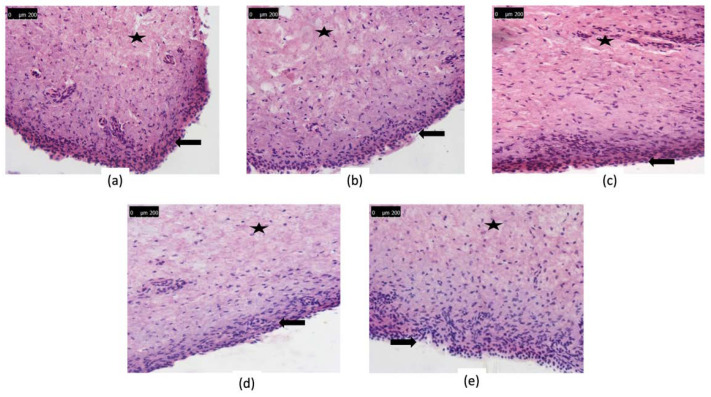
Microscopic images of bladder mucosa treated with different formulations. (**a**) Control, (**b**) St-QCT-SLNs-2, (**c**) C-St-QCT-SLNs-2, (**d**) G_9_St-QCT-SLNs-2, (**e**) G_10_C-St-QCT-SLNs-2. Urothelium 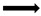 and lamina propria 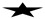. Abbreviations: G_9_: carbapol/poloxamer 407 gel, G_10_: chitosan/poloxamer 407 gel, QCT: quercetin, St: stearic acid, C: coated, SLNs: solid lipid nanoparticles.

A histopathological evaluation of bladder mucosa after treatment with either SLNs alone (St-QCT-SLNs-2 and C-St-QCT-SLNs-2) or gel loaded SLNs (G_9_St-QCT-SLNs-2 and G_10_C-St-QCT-SLNs-2) versus control (untreated) bladder mucosa was carried out using a light microscope. Urinary bladder mucosa treated with different formulations are shown in Figure 7. The urothelial barrier was seen to be intact, as indicated by the presence of tightly packed hexagonal-shaped umbrella cells. The urothelium and the lamina propria showed no change in morphology as compared to control bladder mucosa. These suggest that formulations instillation did not induce any damage to the bladder tissues. Therefore, the developed SLNs and SLNs loaded in situ gels might be regarded as safe [35].

## 4. Conclusions

The goal of the research was to develop an intravesical drug delivery system to enhance the therapeutic efficacy of the delivered drugs against bladder cancer and overcome the shortcomings of the systemic delivery by direct local delivery to the bladder. For this purpose, an intravesical delivery system made of mucoadhesive in situ gel loaded with cationic SLNs was prepared and characterized. QCT was employed as a model drug as it demonstrated high anti-cancer potential against various cell lines; however, few research highlighted its activity against bladder cancer. The results were promising as the QCT loaded cationic nanoparticles were able to achieve highly sustained release profile, as well as high cytotoxicity against T-24 human bladder cancer cells. The developed system showed high mucoadhesion potential to the bladder mucosa after irrigation with artificial urine, good penetration into the bladder wall, as well as short term safety on the bladder tissue. To our knowledge, this is the first SLNs loaded in situ gel as a potential platform for the intravesical management of bladder cancer. However, more animal and clinical studies are required to investigate the possibility of its implementation in clinical practice.

## Figures and Tables

**Table 1:** The composition and characterization parameters of different SLNs formulations.

Formulation Code	Lipid Type	Lipid to QCT Ratio	Stabilizer Type	Stabilizer Concentration (%*w*/*v*)	PS (nm)	PDI	ZP (mV)	EE (%)	DL (%)
**Pr-QCT-SLNs-1**	Precirol	15:1	Tween 80	1.25	182 ± 3	0.33 ± 0.01	-	65 ± 1.2	-
**Pr-QCT-SLNs-2**	Precirol	15:1	Tween 80	2.5	131 ± 2	0.27 ± 0.02	-	60.3 ± 1	-
**Pr-QCT-SLNs-3**	Precirol	15:1	Tween 80	5	114 ± 2	0.22 ± 0.01	-	45.6 ± 1.5	-
**Pr-QCT-SLNs-4**	Precirol	30:1	P188	0.1	691 ± 14	0.38 ± 0.07	-	98.2 ± 0.6	-
**Pr-QCT-SLNs-5**	Precirol	30:1	P188	0.5	341 ± 3	0.18 ± 0.01	-	98.4 ± 0.6	-
**Pr-QCT-SLNs-6**	Precirol	30:1	P188	0.75	364 ± 9	0.25 ± 0.01	-	98.8 ± 0.8	-
**Pr-QCT-SLNs-7**	Precirol	5:1	P188	0.5	346 ± 21	0.46 ± 0.04	-	98.6 ± 1	19.2 ± 1
**Pr-QCT-SLNs-8**	Precirol	10:1	P188	0.5	220 ± 2	0.32 ± 0.02	−33 ± 7	99 ± 1.1	8 ± 0.5
**Pr-QCT-SLNs-9**	Precirol	15:1	P188	0.5	304 ± 1	0.33 ± 0.02	-	98.4 ± 0.7	5.5 ± 0.4
**Pr-SLNs-8**	Precirol	10:1	P188	0.5	264 ± 4	0.25 ± 0.02	-	-	-
**C-Pr-QCT-SLNs-8**	Precirol	10:1	P188	0.5	243 ± 3	0.33 ± 0.03	20 ± 4	98.4 ± 1	6.7 ± 0.4
**St-QCT-SLNs-1**	Stearic acid	5:1	P188	0.5	323 ± 7	0.32 ± 0.03	-	98 ± 0.7	18.1 ± 1.1
**St-QCT-SLNs-2**	Stearic acid	10:1	P188	0.5	262 ± 7	0.23 ± 0.01	−34 ± 7	98.3 ± 1.5	8.87 ± 0.3
**St-QCT-SLNs-3**	Stearic acid	15:1	P188	0.5	359 ± 7	0.19 ± 0.02	−37 ± 7	98 ± 1	6.27 ± 0.1
**St-SLNs-2**	Stearic acid	10:1	P188	0.5	240 ± 5	0.13 ± 0.03	-	-	-
**C-St-QCT-SLNs-2**	Stearic acid	10:1	P188	0.5	275 ± 3	0.21 ± 0.02	29 ± 6	99 ± 1.2	8.85 ± 0.6

Results are presented as mean ± standard deviation (*n* = 3). Abbreviations: PS: particle size, PDI: polydispersity index, ZP: zeta potential, EE: entrapment efficiency, DL: drug loading, P188: poloxamer 188, QCT: quercetin, Pr: precirol, St: stearic acid, C: coated, SLNs: solid lipid nanoparticles.

**Table 2:** The release kinetics of QCT from different SLNs formulations.

Formulation Code	Zero-Order	First-Order	Hixson-Crowell	Higuchi	Korsmeyer-Peppas	
	r^2^	r^2^	r^2^	r^2^	r^2^	n
**Pr-QCT-SLNs-7**	0.901	0.995	0.995	0.975	0.997	0.60
**Pr-QCT-SLNs-8**	0.909	0.996	0.997	0.967	0.998	0.82
**Pr-QCT-SLNs-9**	0.901	0.998	0.995	0.964	0.998	0.62
**C-Pr-QCT-SLNs-8**	0.868	0.981	0.962	0.979	0.995	0.68
**St-QCT-SLNs-2**	0.890	0.993	0.997	0.971	0.998	0.78
**C-St-QCT-SLNs-2**	0.860	0.996	0.997	0.967	0.999	0.81

Abbreviations: QCT: quercetin, Pr: precirol, St: stearic acid, C: coated, SLNs: solid lipid nanoparticles. The numbers 7,8 and 9 donate lipid to drug ratios 5:1, 10:1 and 15:1 respectively.

**Table 3:** The storage stability of different SLNs.

Formulation Code	Colloidal Properties and %EE	Zero Time	One Month	Three Months
**Pr-QCT-SLNs-8**	Particle size (nm)	220 ± 2	204 ± 3	225 ± 5
	PDI	0.32 ± 0.02	0.38 ± 0.02	0.28 ± 0.02
	EE (%)	99 ± 1.1	98.8 ± 1.2	98.5 ± 1.5
**C-Pr-QCT-SLNs-8**	Particle size (nm)	243 ± 3	255 ± 5	243 ± 3
	PDI	0.33 ± 0.03	0.35 ± 0.02	0.28 ± 0.003
	EE (%)	98.4 ± 1	97.9 ± 1.2	98 ± 2
**St-QCT-SLNs-2**	Particle size (nm)	262 ± 7	287 ± 5	297 ± 3
	PDI	0.23 ± 0.01	0.33 ± 0.03	0.27 ± 0.01
	EE (%)	98.3 ± 1.5	98 ± 1	98.5 ± 1.2
**C-St-QCT-SLNs-2**	Particle size (nm)	275 ± 8	292 ± 3	311 ± 6
	PDI	0.21 ± 0.03	0.27 ± 0.002	0.24 ± 0.01
	EE (%)	99 ± 1.2	98.2 ± 1	98.6 ± 0.9

Results are presented as mean ± standard deviation (*n* = 3). Abbreviations: PDI: polydispersity index, EE: entrapment efficiency, QCT: quercetin, Pr: precirol, St: stearic acid, C: coated, SLNs: solid lipid nanoparticles.

**Table 4:** The DSC parameters of heating curves for different SLNs and their components.

Sample	Peak Onset (°C)	Melting Point (°C)	Enthalpy (J/g)
**QCT**	75.75	117.29	−159.64
**QCT**	305.17	317.6	−100.81
**QCT**	345.23	356.16	+131.47
**Precirol**	47.2	64.28	−175.2
**Pr-QCT-SLNs-8**	45.1	63.66	−67.5
**Stearic acid**	46	56.06	−196
**St-QCT-SLNs-2**	44.3	54.5	−76.87
**C-St-QCT-SLNs-2**	43.2	53.04	−54.6
**Chitosan**	273.83	310.33	+204.17

Abbreviations: QCT: quercetin, Pr: precirol, St: stearic acid, C: coated, SLNs: solid lipid nanoparticles.

**Table 5:** Composition and physical characteristics of different P407 based in situ gels.

Gel Code	Poloxamers		Mucoadhesive Polymers	Gelation Temp. (°C)	Gelation Time (s)	Erosion Time (h)	Mucoadhesion	% Gel Pushed through Catheter
	P407 (%*w*/*w*)	P188 (%*w*/*w*)						
**G_1_**	15	-	-	30.7 ± 0.75	30 ± 3.25	3.5 ± 0.25	NA	-
**G_2_**	16	-	-	29 ± 0.25	30 ± 2	6 ± 0.25	NA	-
**G_3_**	18	-	-	26 ± 0.3	25 ± 3	10 ± 0.25	NA	-
**G_4_**	25	-	-	22.1 ± 0.17	10 ± 2.5	48 ± 0.5	NA	-
**G_5_**	20	-	-	25 ± 0.15	11 ± 2	20.5 ± 0.25	NA	-
**G_6_**	20	2.5	-	27 ± 0.2	20 ± 2.5	18 ± 0.5	NA	-
**G_7_**	20	5	-	31 ± 0.15	24 ± 3	15 ± 0.25	NA	-
**G_8_**	20	-	HPMC (1%)	20 ± 0.25	7 ± 1	-	NA	-
**G_9_**	20	-	Cb (1%)	24.7 ± 0.3	9.6 ± 1.5	23 ± 0.25	Good	96.1 ± 0.7
**G_9_St-QCT-SLNs-2**	20	-	Cb (1%)	24.7 ± 0.15	10.3 ± 1.5	24 ± 0.25	Good	96.53 ± 1.1
**G_10_**	20	-	Cs (1%)	23.5 ± 0.25	8.3 ± 1.25	23.5 ± 0.25	Good	96.42 ± 1
**G_10_C-St-QCT-SLNs-2**	20	-	Cs (1%)	23 ± 0.11	8 ± 1	27 ± 0.25	Good	95.59 ± 0.8

Results are presented as mean ± standard deviation (*n* = 3). Abbreviations: P407: poloxamer 407, P188: poloxamer 188, HPMC: hydroxy propyl methyl cellulose, Cb: carbopol 974P, Cs: chitosan, G: gel, G_9_: carbapol/poloxamer 407 gel, G_10_: chitosan/poloxamer 407 gel, QCT: quercetin, St: stearic acid, C: coated, SLNs: solid lipid nanoparticles, NA: Not available.

## Data Availability

Not applicable.
